# (11-Methyl­pyrido[2,3-*b*][1,4]benzo­diazepin-6-yl)(phen­yl)methanone

**DOI:** 10.1107/S1600536810030564

**Published:** 2010-08-11

**Authors:** Fuqiang Shi, Long Zhang, Jin-Feng Wang, Ya-Feng Li

**Affiliations:** a School of Chemical Engineering, Changchun University of Technology, Changchun 130012, People’s Republic of China

## Abstract

In the title compound, C_20_H_15_N_3_O, the diazepine ring adopts a boat conformation. The dihedral angle between pyridine and benzene rings is 55.2 (1)°. The benzoyl phenyl ring forms dihedral angles of 49.4 (1) and 75.9 (1)°, respectively, with the pyridine and benzene rings. In the crystal, mol­ecules are linked into centrosymmetric dimers by pairs of C—H⋯N hydrogen bonds.

## Related literature

For general background to pyridobenzodiazepine derivatives, see: Eberlein *et al.* (1987 ▶); Horton *et al.* (2003 ▶); Shi *et al.* (2008 ▶, 2010 ▶); Tahtaoui *et al.* (2004 ▶). For a related structure, see: Spirlet *et al.* (2003 ▶).
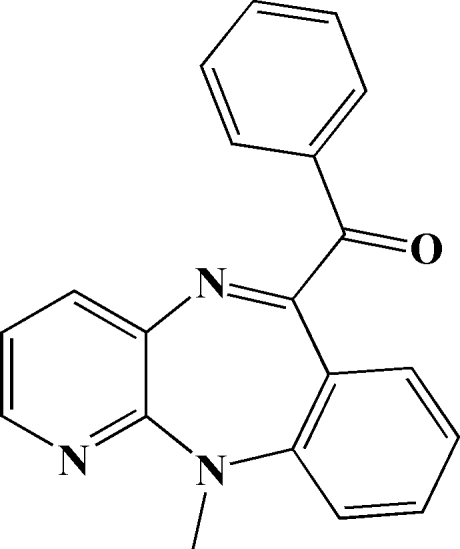


## Experimental

### 

#### Crystal data

C_20_H_15_N_3_O
*M* *_r_* = 313.35
Monoclinic, 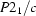

*a* = 8.4442 (17) Å
*b* = 16.503 (3) Å
*c* = 11.682 (2) Å
β = 98.14 (3)°
*V* = 1611.6 (6) Å^3^
*Z* = 4
Mo *K*α radiation
μ = 0.08 mm^−1^
*T* = 293 K
0.37 × 0.30 × 0.19 mm

#### Data collection

Rigaku R-AXIS RAPID diffractometer
Absorption correction: multi-scan (*ABSCOR*; Higashi, 1995 ▶) *T*_min_ = 0.389, *T*_max_ = 0.431
14767 measured reflections
3587 independent reflections
2492 reflections with *I* > 2σ(*I*)
*R*_int_ = 0.048

#### Refinement

*R*[*F* ^2^ > 2σ(*F* ^2^)] = 0.048
*wR*(*F* ^2^) = 0.131
*S* = 1.05
3587 reflections
217 parameters
H-atom parameters constrained
Δρ_max_ = 0.22 e Å^−3^
Δρ_min_ = −0.16 e Å^−3^

### 

Data collection: *PROCESS-AUTO* (Rigaku, 1998 ▶); cell refinement: *PROCESS-AUTO*; data reduction: *CrystalStructure* (Rigaku/MSC, 2002 ▶); program(s) used to solve structure: *SHELXS97* (Sheldrick, 2008 ▶); program(s) used to refine structure: *SHELXL97* (Sheldrick, 2008 ▶); molecular graphics: *DIAMOND* (Brandenburg, 2000 ▶); software used to prepare material for publication: *SHELXL97*.

## Supplementary Material

Crystal structure: contains datablocks I, global. DOI: 10.1107/S1600536810030564/ci5138sup1.cif

Structure factors: contains datablocks I. DOI: 10.1107/S1600536810030564/ci5138Isup2.hkl

Additional supplementary materials:  crystallographic information; 3D view; checkCIF report

## Figures and Tables

**Table 1:** Hydrogen-bond geometry (Å, °)

*D*—H⋯*A*	*D*—H	H⋯*A*	*D*⋯*A*	*D*—H⋯*A*
C15—H15⋯N1^i^	0.93	2.56	3.463 (2)	163

Symmetry code: (i) 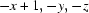.

**Fig. 1.**
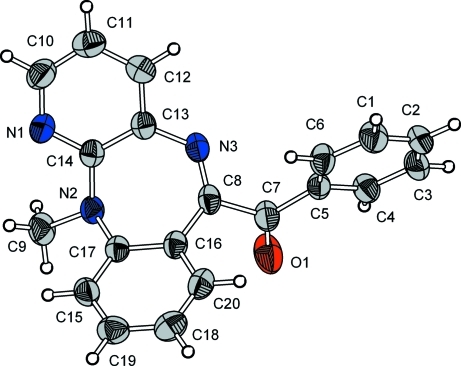
The molecular structure of the title compound, with the atom-labelling scheme. Displacement ellipsoids are shown at the 50% probability level.

## supplementary crystallographic information

### Comment

Pyridobenzodiazepine derivatives possess biological and pharmacological activities (Horton *et al.*, 2003). In most of the reported pyridobenzodiazepines amino or aryl or alkyl group is attached at the C6-position of the heterocyclic nucleus (Eberlein *et al.*, 1987; Tahtaoui *et al.*, 2004; Shi *et al.*, 2008, 2010) while the attachment of a ketone group has not been reported. We report here the crysatal structure of the title compound which contains a benzoyl group at the C6-position.

Bond lengths and angles in the title molecule (Fig.1) are comparable with those observed in a related structure (Spirlet *et al.*, 2003). The diazepine ring displays a boat conformation. The dihedral angle between pyridine and C15-C20 benzene rings is 55.2 (1)°. The benzoyl phenyl ring forms dihedral angles of 49.4 (1)° and 75.9 (1)°, respectively, with the pyridine and benzene ring of the benzodiazepine ring system.

In the crystal structure, the molecules are linked into dimers by C15—H15···N1 hydrogen bonds (Table 1).

### Experimental

Polyphosphoric acid (254 mg, 0.75 mmol), N-2-methyl-N-2-phenylpyridine-2,3-diamine (100 mg, 0.5 mmol) and 2-phenylacetic acid (102 mg, 0.75 mmol) were dissolved in POCl_3_ (5 ml). The solution was heated at 368 K in an oil bath for 7 h and the solution was poured into ice-water (20 ml), treated with 5 N NaOH to pH 9-10, and then extracted with EtOAc (3 × 20 ml). The combined organic phase was washed with saturated NaHCO_3_ and brine, dried with anhydrous Na_2_SO_4_, concentrated *in vacuo*, and purified by flash chromatography with petroleum ether/EtOAc (10:1, v/v) as eluent to afford a mixture of 6-benzyl-11-methylpyrido[2,3-b][1,4]benzodiazepine and 6-benzoyl-11-methylpyrido[2,3-b][1,4]benzodiazepine. The mixture was dissolved in dichloromethane (5 ml), stirred for 24 h under oxygen at room temperature and 6-benzyl-11-methylpyrido[2,3-b][1,4]benzodiazepine disappeared. The reagent was concentrated *in vacuo*, purified by flash chromatography (yield: 140 mg, 95%) and then crystallized from dichloromethane to obtain colourless crystals of the title compound suitable for X-ray analysis.

### Refinement

H atoms were positioned geometrically [C–H = 0.93–0.96 Å] and treated as riding with *U*_iso_(H) = 1.5U_eq_(C_methyl_) and 1.2U_eq_(C).

### Crystal data

**Table:**

C_20_H_15_N_3_O	*F*(000) = 656
*M**_r_* = 313.35	*D*_x_ = 1.291 Mg m^−^^3^
Monoclinic, *P*2_1_/*c*	Mo *K*α radiation, λ = 0.71073 Å
Hall symbol: -P 2ybc	Cell parameters from 2000 reflections
*a* = 8.4442 (17) Å	θ = 3.0–27.5°
*b* = 16.503 (3) Å	µ = 0.08 mm^−^^1^
*c* = 11.682 (2) Å	*T* = 293 K
β = 98.14 (3)°	Block, yellow
*V* = 1611.6 (6) Å^3^	0.37 × 0.30 × 0.19 mm
*Z* = 4	

### Data collection

**Table:**

Rigaku R-AXIS RAPID diffractometer	3587 independent reflections
Radiation source: fine-focus sealed tube	2492 reflections with *I* > 2σ(*I*)
graphite	*R*_int_ = 0.048
Detector resolution: 10.00 pixels mm^-1^	θ_max_ = 27.5°, θ_min_ = 3.0°
ω scans	*h* = −10→9
Absorption correction: multi-scan (*ABSCOR*; Higashi, 1995)	*k* = −21→21
*T*_min_ = 0.389, *T*_max_ = 0.431	*l* = −14→15
14767 measured reflections	

### Refinement

**Table:**

Refinement on *F*^2^	Primary atom site location: structure-invariant direct methods
Least-squares matrix: full	Secondary atom site location: difference Fourier map
*R*[*F*^2^ > 2σ(*F*^2^)] = 0.048	Hydrogen site location: inferred from neighbouring sites
*wR*(*F*^2^) = 0.131	H-atom parameters constrained
*S* = 1.05	*w* = 1/[σ^2^(*F*_o_^2^) + (0.0594*P*)^2^ + 0.2193*P*] where *P* = (*F*_o_^2^ + 2*F*_c_^2^)/3
3587 reflections	(Δ/σ)_max_ = 0.001
217 parameters	Δρ_max_ = 0.22 e Å^−^^3^
0 restraints	Δρ_min_ = −0.16 e Å^−^^3^

### Special details

**Table:**

Geometry. All e.s.d.'s (except the e.s.d. in the dihedral angle between two l.s. planes) are estimated using the full covariance matrix. The cell e.s.d.'s are taken into account individually in the estimation of e.s.d.'s in distances, angles and torsion angles; correlations between e.s.d.'s in cell parameters are only used when they are defined by crystal symmetry. An approximate (isotropic) treatment of cell e.s.d.'s is used for estimating e.s.d.'s involving l.s. planes.
Refinement. Refinement of *F*^2^ against ALL reflections. The weighted *R*-factor *wR* and goodness of fit *S* are based on *F*^2^, conventional *R*-factors *R* are based on *F*, with *F* set to zero for negative *F*^2^. The threshold expression of *F*^2^ > σ(*F*^2^) is used only for calculating *R*-factors(gt) *etc*. and is not relevant to the choice of reflections for refinement. *R*-factors based on *F*^2^ are statistically about twice as large as those based on *F*, and *R*- factors based on ALL data will be even larger.

### Fractional atomic coordinates and isotropic or equivalent isotropic displacement parameters (Å^2^)

**Table:**

	*x*	*y*	*z*	*U*_iso_*/*U*_eq_	
O1	0.84497 (15)	0.36871 (8)	−0.05524 (17)	0.0774 (5)	
N2	0.66756 (14)	0.13174 (8)	0.03808 (12)	0.0394 (3)	
N3	0.99137 (15)	0.19569 (8)	0.04732 (13)	0.0432 (3)	
C1	1.3716 (2)	0.31549 (11)	−0.12859 (17)	0.0529 (5)	
H1	1.4411	0.2799	−0.1580	0.063*	
C2	1.4173 (2)	0.39435 (11)	−0.10549 (16)	0.0497 (4)	
H2	1.5176	0.4119	−0.1192	0.060*	
C3	1.3153 (2)	0.44719 (11)	−0.06219 (16)	0.0500 (5)	
H3	1.3468	0.5004	−0.0457	0.060*	
C4	1.16610 (19)	0.42134 (10)	−0.04322 (16)	0.0464 (4)	
H4	1.0970	0.4575	−0.0146	0.056*	
C5	1.11770 (18)	0.34200 (9)	−0.06626 (14)	0.0387 (4)	
C6	1.2226 (2)	0.28871 (10)	−0.10835 (16)	0.0472 (4)	
H6	1.1930	0.2350	−0.1230	0.057*	
C7	0.9509 (2)	0.31859 (10)	−0.05454 (17)	0.0468 (4)	
C8	0.90799 (18)	0.23004 (9)	−0.03891 (15)	0.0402 (4)	
C9	0.5167 (2)	0.11410 (12)	0.08085 (17)	0.0526 (5)	
H9A	0.4339	0.1483	0.0421	0.079*	
H9B	0.5284	0.1241	0.1626	0.079*	
H9C	0.4887	0.0583	0.0660	0.079*	
C10	0.9020 (2)	−0.02679 (11)	0.18587 (17)	0.0571 (5)	
H10	0.8833	−0.0755	0.2217	0.069*	
C11	1.0573 (2)	−0.00296 (11)	0.18578 (16)	0.0536 (5)	
H11	1.1418	−0.0357	0.2177	0.064*	
C12	1.0848 (2)	0.07088 (11)	0.13706 (15)	0.0478 (4)	
H12	1.1890	0.0897	0.1390	0.057*	
C13	0.95740 (19)	0.11717 (9)	0.08517 (14)	0.0391 (4)	
C14	0.80223 (18)	0.08588 (9)	0.08663 (13)	0.0376 (4)	
N1	0.77531 (18)	0.01626 (8)	0.13702 (13)	0.0494 (4)	
C15	0.53710 (19)	0.11522 (10)	−0.16328 (16)	0.0447 (4)	
H15	0.4594	0.0820	−0.1390	0.054*	
C16	0.77505 (18)	0.19642 (9)	−0.12099 (14)	0.0383 (4)	
C17	0.65890 (17)	0.14649 (9)	−0.08261 (14)	0.0358 (3)	
C18	0.6450 (2)	0.18248 (12)	−0.31775 (16)	0.0563 (5)	
H18	0.6406	0.1941	−0.3960	0.068*	
C19	0.5310 (2)	0.13338 (11)	−0.27957 (16)	0.0530 (5)	
H19	0.4489	0.1122	−0.3326	0.064*	
C20	0.7655 (2)	0.21400 (11)	−0.23854 (16)	0.0497 (4)	
H20	0.8419	0.2476	−0.2639	0.060*	

### Atomic displacement parameters (Å^2^)

**Table:**

	*U*^11^	*U*^22^	*U*^33^	*U*^12^	*U*^13^	*U*^23^
O1	0.0463 (7)	0.0440 (8)	0.1469 (16)	0.0030 (6)	0.0313 (8)	0.0024 (8)
N2	0.0350 (7)	0.0437 (8)	0.0418 (7)	−0.0032 (5)	0.0136 (6)	0.0033 (6)
N3	0.0388 (7)	0.0397 (8)	0.0519 (9)	−0.0071 (6)	0.0095 (6)	−0.0011 (6)
C1	0.0422 (9)	0.0514 (11)	0.0686 (12)	0.0050 (8)	0.0196 (9)	0.0014 (9)
C2	0.0360 (9)	0.0570 (11)	0.0557 (11)	−0.0089 (7)	0.0053 (8)	0.0049 (8)
C3	0.0440 (9)	0.0414 (9)	0.0628 (12)	−0.0123 (7)	0.0009 (8)	−0.0029 (8)
C4	0.0409 (9)	0.0368 (9)	0.0616 (11)	0.0001 (7)	0.0076 (8)	−0.0049 (8)
C5	0.0356 (8)	0.0331 (8)	0.0482 (9)	−0.0025 (6)	0.0086 (7)	0.0025 (7)
C6	0.0478 (9)	0.0337 (9)	0.0626 (11)	−0.0027 (7)	0.0171 (8)	−0.0018 (8)
C7	0.0408 (9)	0.0385 (9)	0.0631 (11)	−0.0020 (7)	0.0143 (8)	0.0009 (8)
C8	0.0360 (8)	0.0366 (8)	0.0513 (10)	−0.0042 (6)	0.0175 (7)	−0.0004 (7)
C9	0.0435 (9)	0.0603 (12)	0.0586 (11)	−0.0028 (8)	0.0231 (9)	0.0047 (9)
C10	0.0689 (13)	0.0437 (10)	0.0566 (11)	−0.0028 (9)	0.0016 (10)	0.0092 (8)
C11	0.0584 (11)	0.0482 (10)	0.0516 (11)	0.0088 (8)	−0.0018 (9)	−0.0011 (8)
C12	0.0415 (9)	0.0511 (10)	0.0498 (10)	0.0009 (7)	0.0027 (8)	−0.0041 (8)
C13	0.0405 (8)	0.0392 (9)	0.0384 (8)	−0.0045 (6)	0.0088 (7)	−0.0039 (7)
C14	0.0424 (9)	0.0367 (8)	0.0346 (8)	−0.0035 (6)	0.0088 (7)	−0.0019 (6)
N1	0.0536 (9)	0.0425 (8)	0.0520 (9)	−0.0076 (6)	0.0064 (7)	0.0090 (7)
C15	0.0399 (9)	0.0382 (9)	0.0556 (11)	−0.0039 (7)	0.0056 (8)	−0.0008 (7)
C16	0.0383 (8)	0.0357 (8)	0.0432 (9)	−0.0006 (6)	0.0132 (7)	0.0016 (7)
C17	0.0361 (8)	0.0312 (8)	0.0414 (9)	0.0015 (6)	0.0099 (7)	−0.0003 (6)
C18	0.0690 (12)	0.0573 (12)	0.0423 (10)	0.0052 (9)	0.0075 (9)	0.0054 (8)
C19	0.0570 (11)	0.0507 (11)	0.0477 (10)	0.0012 (8)	−0.0047 (9)	−0.0041 (8)
C20	0.0555 (10)	0.0461 (10)	0.0498 (11)	−0.0017 (8)	0.0156 (9)	0.0083 (8)

### Geometric parameters (Å, °)

**Table:**

O1—C7	1.218 (2)	C9—H9B	0.96
N2—C14	1.416 (2)	C9—H9C	0.96
N2—C17	1.422 (2)	C10—N1	1.342 (2)
N2—C9	1.462 (2)	C10—C11	1.369 (3)
N3—C8	1.277 (2)	C10—H10	0.93
N3—C13	1.412 (2)	C11—C12	1.379 (3)
C1—C2	1.373 (2)	C11—H11	0.93
C1—C6	1.385 (2)	C12—C13	1.387 (2)
C1—H1	0.93	C12—H12	0.93
C2—C3	1.372 (3)	C13—C14	1.411 (2)
C2—H2	0.93	C14—N1	1.325 (2)
C3—C4	1.377 (2)	C15—C19	1.385 (3)
C3—H3	0.93	C15—C17	1.392 (2)
C4—C5	1.387 (2)	C15—H15	0.93
C4—H4	0.93	C16—C20	1.395 (2)
C5—C6	1.387 (2)	C16—C17	1.402 (2)
C5—C7	1.485 (2)	C18—C20	1.377 (3)
C6—H6	0.93	C18—C19	1.380 (3)
C7—C8	1.523 (2)	C18—H18	0.93
C8—C16	1.477 (2)	C19—H19	0.93
C9—H9A	0.96	C20—H20	0.93
			
C14—N2—C17	114.46 (13)	N1—C10—C11	123.60 (17)
C14—N2—C9	116.49 (13)	N1—C10—H10	118.2
C17—N2—C9	116.60 (13)	C11—C10—H10	118.2
C8—N3—C13	122.74 (13)	C10—C11—C12	118.14 (16)
C2—C1—C6	120.38 (17)	C10—C11—H11	120.9
C2—C1—H1	119.8	C12—C11—H11	120.9
C6—C1—H1	119.8	C11—C12—C13	120.11 (16)
C3—C2—C1	120.07 (16)	C11—C12—H12	119.9
C3—C2—H2	120.0	C13—C12—H12	119.9
C1—C2—H2	120.0	C12—C13—C14	117.22 (15)
C2—C3—C4	119.92 (16)	C12—C13—N3	117.58 (14)
C2—C3—H3	120.0	C14—C13—N3	124.75 (14)
C4—C3—H3	120.0	N1—C14—C13	122.73 (15)
C3—C4—C5	120.82 (16)	N1—C14—N2	117.57 (14)
C3—C4—H4	119.6	C13—C14—N2	119.61 (14)
C5—C4—H4	119.6	C14—N1—C10	118.11 (15)
C4—C5—C6	118.82 (15)	C19—C15—C17	120.35 (16)
C4—C5—C7	119.02 (15)	C19—C15—H15	119.8
C6—C5—C7	121.96 (15)	C17—C15—H15	119.8
C1—C6—C5	119.96 (16)	C20—C16—C17	119.45 (15)
C1—C6—H6	120.0	C20—C16—C8	119.57 (15)
C5—C6—H6	120.0	C17—C16—C8	120.98 (14)
O1—C7—C5	121.87 (15)	C15—C17—C16	118.99 (15)
O1—C7—C8	117.75 (15)	C15—C17—N2	122.48 (14)
C5—C7—C8	120.38 (14)	C16—C17—N2	118.51 (14)
N3—C8—C16	128.95 (14)	C20—C18—C19	119.19 (17)
N3—C8—C7	113.99 (14)	C20—C18—H18	120.4
C16—C8—C7	117.03 (14)	C19—C18—H18	120.4
N2—C9—H9A	109.5	C18—C19—C15	120.88 (17)
N2—C9—H9B	109.5	C18—C19—H19	119.6
H9A—C9—H9B	109.5	C15—C19—H19	119.6
N2—C9—H9C	109.5	C18—C20—C16	121.15 (17)
H9A—C9—H9C	109.5	C18—C20—H20	119.4
H9B—C9—H9C	109.5	C16—C20—H20	119.4

### Hydrogen-bond geometry (Å, °)

**Table:**

*D*—H···*A*	*D*—H	H···*A*	*D*···*A*	*D*—H···*A*
C15—H15···N1^i^	0.93	2.56	3.463 (2)	163

Symmetry codes: (i) −*x*+1, −*y*, −*z*.
